# No sweat: African American adolescent girls’ opinions of hairstyle choices and physical activity

**DOI:** 10.1186/s40608-016-0111-7

**Published:** 2016-07-01

**Authors:** Susan J. Woolford, Carole J. Woolford-Hunt, Areej Sami, Natalie Blake, David R. Williams

**Affiliations:** Child Health Evaluation and Research (CHEAR) Unit, Department of Pediatrics, University of Michigan, 300 NIB, Room 6D20, Campus Box 0456, Ann Arbor, MI 48109-5456 USA; Department of Graduate Psychology & Counseling, Andrews University, Berrien Springs, MI USA; Department of Society, Human Development and Health, Harvard University, Cambridge, MA USA

**Keywords:** Physical activity, Adolescents, African American, Disparities, Ethnic identity

## Abstract

**Background:**

Obesity prevalence is higher among African American adolescent (AAA) girls than among non-black girls. Lower levels of physical activity (PA) likely contribute to this disparity; this may be impacted by hairstyle concerns.

**Methods:**

In 2011, focus groups were conducted with AAA girls 14-17 years old (*n* = 36) in Michigan (*n* = 9), California (*n* = 11), and Georgia (*n* = 16). Groups addressed perceptions of hairstyles, exercise, and relationships between the two. Groups were recorded, transcripts reviewed, and themes identified. Adolescents completed a standardized ethnic identity (EI) measure and a survey addressing demographics and PA. Linear regression was used to examine associations between self-reported activity and participants’ characteristics.

**Results:**

Four themes emerged: 1) between ages 8 and 15, when concerns about hairstyles began, participants changed from “juvenile” (natural) styles to “adult” (straightened) styles; 2) participants avoided getting wet or sweating during exercise because their straightened hair became “nappy;” 3) braids with extensions and natural styles were viewed as better for exercise but not very attractive; 4) participants almost universally selected long, straight hairstyles as most attractive. In Michigan and California, EI was positively associated with levels of PA (*p* < 0.05) and overall having extensions was also positively associated with levels of PA.

**Conclusions:**

A preference for straight hair may contribute to AAA girls avoiding certain activities due to concerns about sweat affecting their hair. Furthermore, EI and hairstyle choice appear to be associated with levels of PA for some participants. Efforts to increase AAA girls’ PA may benefit from approaches that address hairstyle choices and EI.

## Background

While childhood obesity has affected American children of all races and ethnicities, both genders, and across the gamut of socioeconomic groups, disparities exist in regard to its prevalence in certain subpopulations [1]. Specifically, African American girls have a higher prevalence of obesity compared to their Caucasian peers. Interestingly, the difference becomes more apparent in older age groups. Among 2–5 year olds, Caucasian and African American girls have a prevalence of obesity of 12 % and 11.7 % respectively and by 6–11 years of age a small difference is noted (17.4 vs. 21.1 %). However, by adolescence a marked difference is evident with a prevalence of 14.5 % vs. 29.2 % respectively for 12 to 19 year olds [1].

Obesity in adolescence is associated with increased risk of numerous illnesses including metabolic syndrome, non-alcoholic fatty liver disease, polycystic ovarian syndrome, orthopedic problems and psychosocial concerns [2]. Obesity in childhood is known to track to adulthood and to put those affected at risk for developing a number of comorbid conditions such as type 2 diabetes, cardiovascular disease, and a number of cancers [2–5]. While 36.3 % of adult American women overall are obese, for African American women, the prevalence of obesity is 58.6 % [6]. They are also known to have higher rates of obesity-related comorbidities such as hypertension and stroke than their peers [7]. Finding ways to address this issue early in the life course could have significant public health effects.

Although the reasons for the disparity in the prevalence of obesity among African American females compared to their peers are not completely understood, differences in levels of physical activity appear to play a role. African American adolescent girls have been shown to engage in lower levels of physical activity, to be less likely to participate in organized sports, and to be less likely to participate in gym classes than their peers [8, 9]. It is therefore important to explore the reasons for these lower levels of activity and to identify modifiable factors that might serve as targets for interventions.

Prior studies have explored the barriers to physical activity among African American adolescent girls and women. These barriers include, not having enjoyable options, having a schedule that is too busy, and concerns about hair maintenance [10–13]. However, these studies did not fully explore among adolescent populations the issues that influence hairstyle choices, strategies to promote physical activity in relation to hairstyle choices, or associations between respondent characteristics and their level of physical activity.

### African American women and hair

Work in the social sciences among adult African American women suggests that hair and hairstyle choices have meaning in two important domains, acceptability and quality of hair [12, 13]. For example, there is often the idea that “good hair” which typically refers to straighter, longer hair, is viewed as the best quality hair and is therefore desirable [14]. In addition, there is the perception that to be accepted in mainstream America, particularly in corporate America, straighter hair is more appropriate, whereas braided, locked, or natural (afro) hair is less likely to be viewed as acceptable [14]. In response to these cultural pressures, it is thought that black women often select hairstyles that require hair weaving or some form of straightening process [14]. However, the straightening process is reversed by exposure to moisture (e.g., sweat or rain). Little is known about the role of hair maintenance in physical activity among black adolescent females, and how this may be impacted by ethnic identity.

### Ethnic identity and hairstyle choices

Ethnic identity, defined as one’s sense of self as a member of an ethnic group, has been shown to be related to lifestyle choices, esthetic preferences, and hairstyle choices among adults [12–16]. Ethnic identity with regards to hairstyle choice has not been explored among adolescents.

### Study hypothesis and objective

We hypothesized that 1) higher ethnic identity, and 2) hairstyle choices that do not require straightening, might be associated with higher levels of physical activity. As a first step to explore these questions and in an effort to inform interventions to increase physical activity among African American adolescent girls, our objective was to explore AAA girls’ perceptions about hairstyle choices and physical activity, including strategies for increasing activity. In addition, we wished to examine whether an association exists between ethnic identity and levels of physical activity.

## Methods

### Study design

In this mixed-methods, multi-site study, the qualitative data were elicited from focus groups and the quantitative data were collected from cross-sectional survey data. Due to the presence of only limited data on this topic among AAA girls, a hypothesis-generating qualitative approach utilizing focus groups was used to explore participants’ perspectives regarding hairstyle choices and physical activity. The study protocol was approved by the Institutional Review Board of the University of Michigan Medical School. Written informed consent was obtained from parents/guardians and written assent from the adolescents.

### Recruitment

#### Sites

In Summer 2011 a convenience group of churches from Michigan, Georgia and California were contacted to participate in the study. The churches all had mainly African American memberships and to our knowledge memberships that were socioeconomically diverse. *Approach* – Letters were sent to church administrators describing the study and requesting their approval to conduct the study at their site. Churches posted flyers in their communities, inviting African American girls between 14 and 17 years of age to participate in the study. A telephone number was provided for parents of the interested teens to call. Upon contacting our research center, participants’ age, race, gender and fluency in English were confirmed and the details of the study explained. Those who expressed a desire to participate were added to the list for the focus group closest to their home. The consent/assent process occurred in person (with parents and adolescents present) prior to the focus groups. Adolescent girls participated in the 2-hour focus group sessions without their parent/guardian present. Adolescents were compensated with a $30 gift card for participation.

### Data collection

**Fig. 1:**
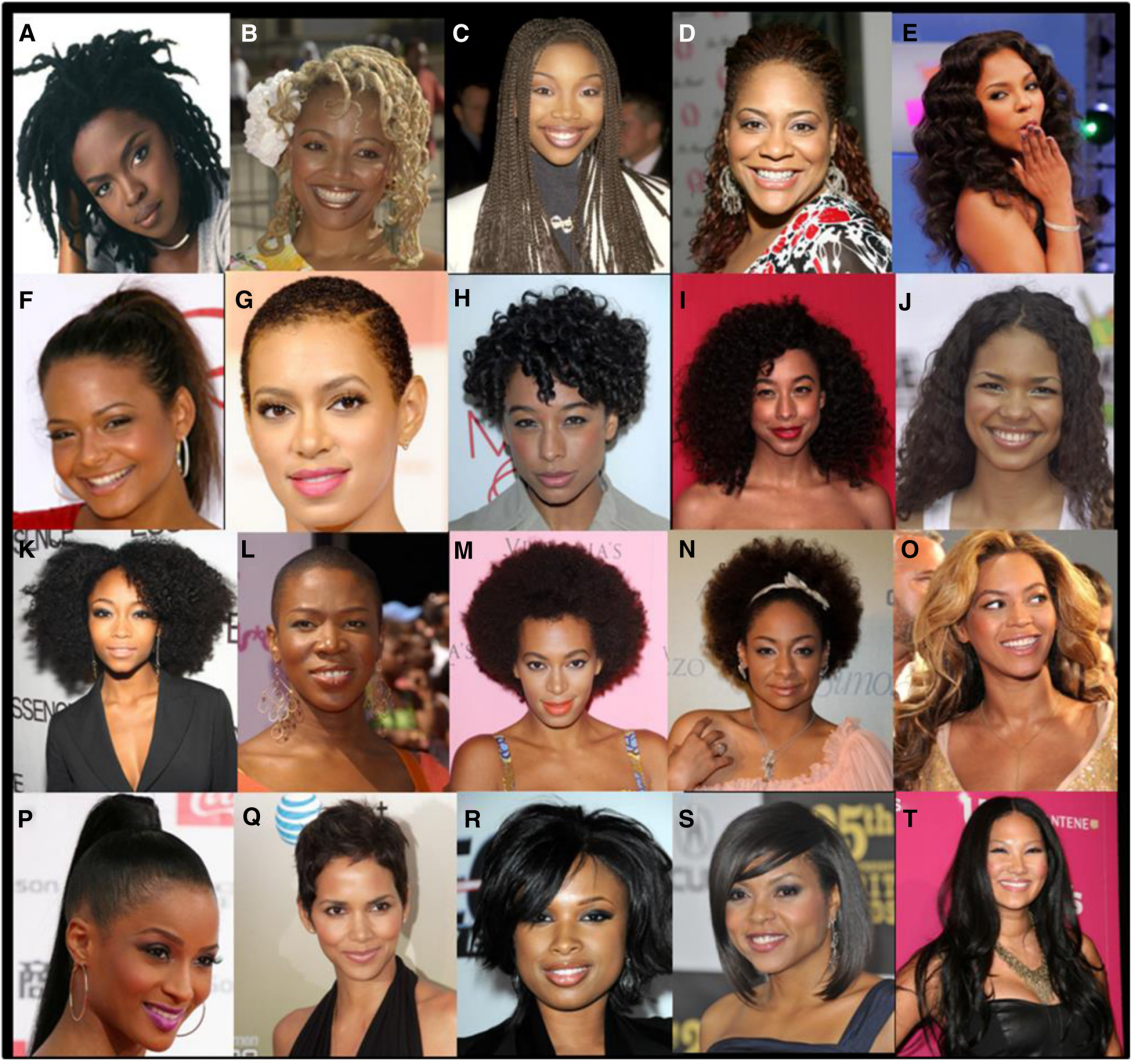
Photo Array of Hairstyles. Copyright permission to publish images was obtained from Getty’s Images, Shutter Stock and PR Photos
  - Anthropometric Measurements – Weight was measured via an electronic scale and height was obtained using a portable stadiometer.
  - Focus Group – The groups were conducted by experienced facilitators (of the same ethnic background and gender of the participants – one with chemically straightened hair and the other with natural hair), Woolford and Woolford-Hunt (an MD employed as the Medical Director of a Pediatric Weight Management Center and a PhD employed as an Associate Professor of Psychology respectively). A guide was used which included prompts to explore barriers to engaging in physical activity, the role of hairstyle choices in physical activity, and preferences for particular hairstyles. The primary questions used in the focus guide are presented in Table 1.

Each session included three components:

1. Survey Completion – Participants completed brief surveys, addressing:
  - Ethnic Identity – Was measured for participants using the Multigroup Ethnic Identity Measure (MEIM) [17], a standardized measure of ethnic identity addressing the following domains: positive ethnic attitudes and sense of belonging, ethnic identity achievement, and ethnic behaviors or practices. This fifteen item instrument that has been validated in a number of ethnic groups including African Americans, contains two types of questions: a) 12 items ask respondents to use a 4 point Likert scale (1 = strongly disagree to 4 = strongly agree) to rate their level of agreement with statements such as “*I have spent time trying to find out more about my ethnic group, such as its history, traditions, and customs*.”; b) 3 items ask respondents to indicate their own and their parents ethnicity [18, 19]. Thus scores may range from 12 (the lowest level possible score) to 48 (the highest possible score); with higher scores representing a stronger ethnic identity.
  - Physical Activity – Was explored using the five items addressing sports participation and physical activity habits from the previously validated Youth Risk Behavior Survey [20]. Mean number of days per week on which participants achieved at least 60 min of physical activity was calculated from responses to the question: “*During the past 7 days, on how many days were you physically active for a total of at least 60 min per day? (Add up all the time you spent in any kind of physical activity that increased your heart rate and made you breathe hard some of the time.).”*
  - Demographic Data – Were elicited via items addressing age, race, ethnic background, self-reported height and weight.
  - Hair Characteristics, Styles and Preferences – In this novel exploration, picture arrays depicting a variety of hairstyles developed for this study were used to help participants to report characteristics of their hair, and their hairstyle preferences (Examples of similar hairstyles presented in Fig. 1). The array of hairstyles was developed by piloting the examples with a variety of African American women and adolescents. Three different arrays were developed; one to assess texture, one length, and one for hairstyle preferences. In the pilot process respondents were asked the following 3 questions: 1) Please select the picture that is the closest match to the texture of your natural hair. 2) Please select the picture that is the closest match to the longest your hair has been without extensions. 3) Please select the picture of the hairstyle you like the most and the hairstyle you like the least. The pictures were revised during the pilot process until respondents reported they had sufficient options for them to answer the questions posed. In the study, participants also responded to the following 2 questions addressing their typical hairstyle: “Do you typically have extensions” and “Is your hair typically A. Natural, B. Chemically straightened (e.g. relaxed, Brazilian/keratin treatment), C. Chemically curled (e.g. Jheri curls) or D. Other.”

**Table 1:** Primary questions for qualitative component of the focus group discussion

Main Topic Area	Questions
Physical activity and hairstyles	Icebreaker: What if any sports or physical activities do you enjoy? Question 1: What gets in the way of you exercising? Follow-up questions: a) Do you ever get concerned about the way you look when you exercise? What concerns do you have? b) What happens to your hair when you exercise? c) Do concerns about the way your hair will look after you exercise ever keep you from exercising?
Preferred hairstyle choices	Question 2: Please think about different hairstyles. Are there any specific styles that you find more appealing/attractive? Can you describe them? Follow-up questions: a) Why do you like those features?
Current and previous hairstyle choices	Question 3: Now please think about your current hairstyle and those you had when you were younger. For those of you who have a different hairstyle now than when you were younger (e.g. straightened, weaves, braids), at what age did you first change your hairstyle? Follow-up questions: a) What influenced that decision? b) What does your hair say about you?/What do you want your hair to say about you?
Suggestions for ways to increase physical activity	Question 4: Do you have any strategies for maintaining your hairstyle intact while you exercise?

### Data management and analysis

#### Qualitative

All groups were audio and video recorded. Transcripts were systematically analyzed and coded independently using the constant comparative method by two authors to identify themes [21]. Differences were discussed and adjudicated by a third author. Within the main topic areas to be explored, common experiences and patterns were identified and refined in an iterative process. Themes were compared across sites. Lists were made of participant comments related to each theme. From these lists, representative comments were selected for the purpose of illustration. There was high convergence of the thoughts expressed between the focus groups performed. The study was terminated when thematic saturation was achieved, which occurred after 5 focus groups were performed (n = 36 adolescents overall; Michigan n = 9, California n = 11, and Georgia n = 16).

#### Quantitative

Descriptive statistics were calculated for age, BMI (from measured heights and weights), and for types of hairstyles reported. Ethnic identity scores were calculated from responses to the MEIM. Mean ethnic identity scores were calculated for each location. Mean days of physical activity per week was calculated as described above. Linear regression (stratified by location) was used to examine associations between self-reported physical activity and ethnic identity for girls in each location. Due to the small sample size of the study and due to similarities in both the quantitative and qualitative data from Michigan and California, these were grouped for this analysis.

## Results

### Participant characteristics

For participants overall (n = 36), the mean age was 15.7 years old and the mean BMI was 24.7 kg/m^2^ (range 16.7 to 52.7 kg/m^2^) which corresponds to a mean BMI z-score of 0.625 (range -2.058 to 2.624). Approximately one-third were either overweight (16.6 %) or obese (16.6 %).

### Qualitative findings

From the qualitative discussion, 4 themes emerged in all groups. These were 1) a preference for straight rather than natural styles, 2) a change from juvenile to adult styles that typically meant changing form natural to straightened styles, 3) the negative effect of sweating on straightened hair, and 4) the benefits of natural hair and braids in regard to exercise.

### Theme 1: Straightened hairstyles preferred over natural ones

When selecting photographs of hairstyles they found most attractive, participants almost universally selected long hairstyles that required heat or chemical straightening, (such as E and O from Fig. 1). While there was an acknowledgement that some short or natural hairstyles could be nice, it was viewed that these were only attractive for certain people whereas longer, straighter styles were attractive for most types of faces and bodies (Table 2). It also seemed that choosing to have natural hair indicated a degree of independence and lack of concern about societal preferences. Conversely, natural hairstyles were generally viewed as the least attractive and associated with negative connotations (e.g., M from Fig. 1) This was illustrated by the following exchange: *Interviewer: “Um so [M], what does [M] say about her?” Georgia Participant 3: “She looks like she doesn’t care.” Georgia Participant 4: “It looks like she is saying 'does my hair look ugly?' ” (laughing)*. This was a view expressed by several participants in all regions.

**Table 2:** Major themes with examples of representative quotes

Themes	Sample quotes
1) A preference for longer straighter styles rather than natural styles	*Interviewer: “So… the most attractive – which one would you choose?”* *Georgia Participant 3: “Either [E] or [T].”* *Georgia Participant 4: “I like [S] or [O]”* *Georgia Participant 5: “I think it depends on the person. Like I like long hair.”*
2) A change occurred for most from juvenile to adult styles that typically meant changing from natural to straightened styles	*Interviewer: “So is there any association with hairstyles and age?”* *Michigan Participant 5: “Yes. When you’re like 5 your hair is like this…(motioning both hands on top of her head, making little pony tail-type look)… But when you’re 14 your hair looks like that (pointing to the rest of the girls with straightened hair – while several participants nod in agreement).”*
3) The negative effect of sweating on straightened hair	*Interviewer: “What happens to your hair when you exercise?”* *Georgia participant 1: “You sweat it out”* *Georgia participant 2: “It puffs up and it’s not cute anymore.”* *Georgia participant 4: “Especially if you wear your regular hair”* *California participant 1: “It gets wild. It gets nappy.”*
4) Benefits of natural hair and braids in regard to exercise	*Interviewer: “What styles allow you to exercise without problems?”* *California Participant 3: “Braids.”* *Interviewer: “Why haven’t you had braids since middle school?”* *California Participant 5: “It’s ugly.”* *California Participant 6: “It just doesn’t feel right anymore. You’re so used to the new hairstyles.”* *California Participant 7: “Yeah, like your hair always being down.”*

### Theme 2: Change from juvenile (natural) to adult (straightened) styles

There was a strong consensus that with advancing age, hairstyles changed. This change occurred both to make it easier to comb their hair and to have a more adult appearance. The age at which the change occurred varied. However, it appeared that in general between ages 8 and 15, participants became concerned about their hairstyle, and changed from juvenile styles to adult styles which typically required straightening (Table 2). For some participants this process of changing to more adult styles started with straightening their hair at home and later to the more expensive option of going to a salon. The following is an illustrative exchange from a Michigan focus group: *Interviewer: “When did you first flat iron?” Participant 5:“Getting it done done [sic][straightened in a salon] - I was 14 but I got my hair done [straighten at home] at 9.”* This transition in hair styles impacted physical activity, specifically swimming was considered something that could be done while young (with natural hair), but was rarely possible once they changed to straightened, more adult styles.

### Theme 3: Effect of sweating on straightened hair

Participants indicated that since becoming more concerned about their hair, they avoided getting their hair too wet during exercise because it made their hair “puffy” or “nappy” (Table 2). This was a view held by those who had natural hair that was heat straightened and by those who chemically straightened their hair. Those with braids noted that at the times when they straightened their hair, they had similar concerns. Even participants who reported exercising indicated having these concerns. Indeed, some respondents stated that though they exercise, they refrain from too much exertion in order to protect their hairstyle.

### Theme 4: Natural and braided hairstyles allow exercise

Participants recognized certain hairstyles as being more amenable to exercise but viewed these styles as not very attractive (Table 2). In general, participants in Georgia were more accepting of natural hairstyles and were willing to have these styles at times. For example, one participant indicated that she keeps her hair braided during the sports season and then takes it out once the season is over and others were willing to “wrap” their hair in order to exercise as that helped maintain weaved hairstyles. Particularly in California, there was a strong preference among participants for straighter styles vs. braids.

### Quantitative findings

#### Physical activity

The mean number of days per week on which participants reported achieving at least 60 min of physical activity, differed by location (Michigan 3.7 days vs. California 4.5 days vs. Georgia 2.9 days, *p* < 0.05)

#### Hairstyle

In response to the question “Is your hair typically A. Natural, B. Chemically straightened (e.g. relaxed, Brazilian/keratin treatment), C. Chemically curled (e.g. Jheri curls) or D. Other”, most participants reported having natural hair (64 %), 28 % reported having chemically straightened hair, and 8 % reported having “other.” In response to the question “Do you typically heat straighten your hair with a) a hot comb, b) a flat iron or c) I do not heat straighten my hair (choose all that apply)”, 19 % used a hot comb, 70 % used a flat iron, 11 % used both. No participants stated that they do not heat straighten their hair.

#### Physical activity and hairstyle

Mean number of days of 60 min or more of physical activity also differed by whether the respondents indicated that they typically had extensions. For those who indicated “yes” to the question “Do you typically have extensions” the mean number of days of physical activity per week was 4.8 days compared to those who answered “no” for whom the mean number of days of activity per week was 2.9 days (*p* = 0.002) (Table 3).

**Table 3:** Mean days of 60 minutes or more of physical activity vs. hairstyle choice (Extensions)

Typically use extensions		Mean	Std. Err.	[95 % Conf.	Interval]
	No	2.92	0.33	2.24	3.60
	Yes	4.83	0.47	3.87	5.80

#### Ethnic identity

The mean ethnic identity score for respondents overall was 37.5 and did not differ significantly by location (Georgia and Michigan 36.3; California 39.0, *p* = 0.15).

#### Ethnic identity and physical activity

Among participants in Michigan and California, there was a statistically significant association between self-reported physical activity and ethnic identity. Specifically, those scoring higher on the ethnic identity scale reported higher levels of physical activity (p = 0.02). For every 10 unit increase in ethnic identity score, there was an approximately 2 day increase (95 % confidence interval 0.33 to 3.2) in self-reported days of physical activity. For Georgia no relationship was seen between these variables.

## Discussion

This is the first study to our knowledge to explore the role of hairstyle choice, ethnic identity and physical activity among African American adolescent girls. We found that concerns about hairstyle maintenance begin to affect the physical activity choices of African American girls just prior to or in early adolescence, with girls deciding not to swim or exercise too vigorously once they made the change from more natural hairstyles and braids to straighter more adult hairstyles. However, we also found that having extensions, and in Michigan and California, having a higher ethnic identity score was associated with reports of higher levels of physical activity. This may suggest areas for further exploration in efforts to increase physical activity and to combat the current levels of obesity among AAA girls. While the focus group participants provided few suggestions for increasing physical activity, these findings from the analysis of ethnic identity and hairstyle choices provide a novel target for future study and potentially an option for tailoring interventions.

The social norm expressed by the adolescents in all of the focus groups was a strong preference for long, straight hair. The almost unanimous belief that such hair types were most attractive and could be worn by anyone (as opposed to natural hair that only looked good on some people), was noteworthy, as was the concern regarding sweating during exercise because of its deleterious effects on heat or chemically straightened hair. This mirrors findings in studies of adult African American women who express similar opinions regarding exercise and hair [11–13, 22]. It is possible that this is a concern shared by many women of different races, but for African American women and girls, the process of straightening hair after exercise may be more extensive and more expensive than for other races and therefore, be a greater barrier to engaging in physical activity. Previous studies show that black women frequent their hairstylist every 2.5 weeks compared to every 6 weeks for white women [23]. With some estimates suggesting that on average, black women spend $1690 per year while their white counterpart spends $433 per year for hair straightening services by a hairstylist [23].

Consequently, it may be more concerning, particularly if a social norm that supports a preference for straightened hair contributes to lower levels of participation in physical activity and potentially higher levels of obesity and obesity-related comorbidities. Finding ways to counteract the possible social pressure to conform to certain norms, may be associated with positive health outcomes for African American women and girls.

One potential protective factor for AAA girls may be the role of ethnic identity. This is the first study to find a statistically significant relationship between ethnic identity and physical activity for AAA girls. Of note, this was found among the participants in Michigan and California, but not those in Georgia. The underlying reasons for this relationship are not known. It may be that strong feelings about an African American identity are associated with a greater emphasis placed on physical activity or health. There is very limited literature to draw on to support this hypothesis and further work is needed to identify whether this plays a role in explaining our findings in this study. An alternative explanation for the association between levels of physical activity and ethnic identity may be that those with higher ethnic identity scores are less influenced by the societal preference for straight hair and more tolerant of natural hairstyle choices or “frizzy” hair. This may result in higher levels of participation in physical activity than their peers. Again, further work is required to explore this possibility. The reasons why this was not found in Georgia are also unknown. It may be due to the small sample size, and therefore larger studies are needed to shed light on this finding.

### Limitations

Our findings need to be considered within the context of some limitations. First, it is a relatively small study, recruited from churches, in three states in America. This may impact generalizability as findings may differ for all African American girls, for African American girls in different parts of the country and for Black girls in other parts of the world. In addition, the participants indicated their preferences for hairstyles from a picture array of celebrities and their choices may reflect preferences for the celebrities rather than the actual hairstyle. However, in this mixed methods study, participants also discussed their preference for longer, straighter hairstyles apart from the picture array, suggesting that this may be an underlying preference reflected in the images they selected as their favorites. Furthermore, our measure of physical activity relied on self-report. Though the questions were from the widely used Youth Risk Behavior Survey, our findings may have differed if objective measures of activity were utilized.

## Conclusion

This study suggests that hair maintenance concerns may begin to affect AAA girls’ thoughts about and participation in physical activity prior to or in early adolescence. Many AAA girls express a strong preference for long, straight hairstyles which are impacted adversely by sweating. In addition, hairstyle choice (extensions vs. no extensions) appears to be associated with levels of physical activity for our respondents overall and ethnic identity appears to be associated with levels of physical activity for some participants. Interventions to increase physical activity among AAA girls generally have not shown significant results to date [24]. Such efforts may be enhanced by adopting approaches that incorporate the issue of hairstyle choice, reinforce African American beauty, and address the role of ethnic identity.

## Abbreviations

AAA, African American adolescent; PA, Physical Activity; EI, Ethnic Identity; MEIM, Multigroup Ethnic Identity Measure
